# Comfort, Energy Efficiency and Adoption of Personal Cooling Systems in Warm Environments: A Field Experimental Study

**DOI:** 10.3390/ijerph14111408

**Published:** 2017-11-17

**Authors:** Yingdong He, Nianping Li, Xiang Wang, Meiling He, De He

**Affiliations:** 1 College of Civil Engineering, Hunan University, Changsha 410082, China; wangxianghd@126.com (X.W.); hz_hml@163.com (M.H.); hdjh2011@163.com (D.H.); 2 Key Laboratory of Building Safety and Energy Efficiency, Hunan University, Ministry of Education, Changsha 410082, China

**Keywords:** thermal comfort, energy efficiency, radiant cooling desk, desk fan, personal cooling, logistic regression

## Abstract

It is well known that personal cooling improves thermal comfort and save energy. This study aims to: (1) compare different personal cooling systems and (2) understand what influences users’ willingness to adopt them. A series of experiments on several types of personal cooling systems, which included physical measurements, questionnaires and feedback, was conducted in a real office environment. The obtained results showed that personal cooling improved comfort of participants in warm environments. Then an improved index was proposed and used to compare different types of personal cooling systems in terms of comfort and energy efficiency simultaneously. According to the improved index, desk fans were highly energy-efficient, while the hybrid personal cooling (the combination of radiant cooling desk and desk fan) consumed more energy but showed advantages of extending the comfortable temperature range. Moreover, if personal cooling was free, most participants were willing to adopt it and the effectiveness was the main factor influencing their willingness, whereas if participants had to pay, they probably refused to adopt it due to the cost and the availability of conventional air conditioners. Thus, providing effective and free personal cooling systems should be regarded as a better way for its wider application.

## 1. Introduction

Personal comfort systems (PCSs) are gaining popularity due to their better comfort and great energy-saving potential, as compared to conventional air conditioning systems [1,2,3,4]. A PCS efficiently cools or heats local body parts of people so as to keep the whole body comfortable [4]. As a result, people have more chances to avoid discomfort stimulations of the non-neutral environment. Such a feature leads to a high energy-efficiency because only a small amount of energy is consumed by a PCS to condition the local body parts rather than the whole indoor environment and the set point temperature range of air conditioning systems (for maintaining the background environment in buildings) is widened. As proved by many studies, extending the set point temperature contributes to great energy conservation in air conditioning system [4,5,6,7].

A personal cooling system is an important part of PCS. In recent years, many studies have been carried out on personal cooling. Huang et al. [8] proposed a new airflow management technique to supply cool airflow for passengers in a car. The local airflow met the individual requirements of passengers in different zones thus satisfying all of them. Cui et al. [9] undertook a field investigation on passengers’ comfort and behaviors on ten air planes. The results indicated that passengers tended to use the personalized nozzles to ameliorate the warm discomfort. Oh et al. [2] conducted a series of experiments on the air-conditioning system which supplied cool air in cars, then they found it saved more than 20% energy. Likewise, through CFD simulation, Ghosh et al. [1] claimed that personal cooling could reduce both the airflow and the cooling load in the automobile thus improving the energy-efficiency of the air-conditioning system.

Besides the application in mobile vehicles and aircrafts, more personal cooling systems and devices have been studied in building environments. Chen et al. [10] simulated the energy performance of a kind of personal ventilation system. They found the best condition was achieved when the supply air of personal ventilation was at 20 °C while the background environment was at 26 °C. Besides, around 10% energy consumption was saved when the indoor temperature increased from 23 to 26 °C. Chakroun et al. [11] tried to add personalized evaporative coolers to a room with chilled ceiling and displacement ventilation. They reported that personal cooling contributed to a 17.5% energy savings at 21 °C since the supply air temperature of displacement ventilation was increased. However, Yang et al. [12] stated that if the personal cooling system consumed too much extra energy, the total energy may increase although the indoor temperature was elevated. Schiavon and Melikov [13] introduced a new index for evaluate the cooing capacity and the energy use of personal fans. This index made it possible to compare the performances of different fans. Watanabe et al. [14] designed a kind of ventilated chair with two small fans which directly cooled buttocks and backs of users. It was then proved through the experiments that the chair made participants feel neutral in the environment at 28 °C. Likely, Boerstra et al. [15] claimed that desk fans could meet 90% acceptable requirement by ASHRAE Standard 55 [16] for occupants in office room as indoor temperature went up to 28 °C [16]. Further, Atthajariyakul and Lertsatittanakorn [5] conducted tests on small fans. They pointed out that the usage of small fans allowed people to have a neutral thermal sensation in the environment at up to 28 °C and it could help save up to 1959.51 GWh/year of electricity in Thailand. Dalewski et al. [17] studied the combination of personal ventilation cooling with displacement ventilation and they found personal ventilation cooling significantly improved thermal comfort in warm environments and most of the participants found the indoor environment acceptable even when it was at a temperature of 29 °C. Similar to the study of Watanabe et al. [14], Pasut et al. [18] combined office chairs and small energy-efficient fans (merely 2 W) to maintain comfort. The obtained results from experiments indicated that subjective comfort was still maintained in the environment at 29 °C. He et al. [19] used small desk fans as complementary cooling for a radiant ceiling system. It was demonstrated that the desk fans could be regarded as an energy-efficient solution for maintaining comfort when the cooling ceiling was unable to fully cool the indoor environment.

Some other researchers have attempted to explore the performance of personal cooling systems in hotter environments. Based on the results from experiments, Zhai et al. [20,21] drew the conclusion that both floor fans and ceiling fans could improve comfort and perceived air quality in the hot environment at the temperature of 30 °C. Huang et al. [22] held the similar opinion that increased air movement by desk fans significantly reduced the warmth sensation when air temperature was as high as 32 °C. Different from most of previous studies, He et al. [23] designed a new type of personal cooling system (a radiant cooling desk) which adopted radiant cooling panels to cool the users. The results showed that most of participants with this system voted on the acceptable side in the environment at 32 °C and the warm sensation was significantly lowered. Moreover, Wang and Song [24] tested four personal cooling strategies (including fans and some wearable devices) in extremely hot environments (36 °C and 40 °C) by using a manikin. They found that using fans and evaporative cooling clothing simultaneously presented the best cooling effect.

As reviewed above, personal cooling is a practical approach to maintaining users’ comfort in warm environments. Nevertheless, fans and personal ventilation are the dominant topic in previous studies while other personal cooling systems account for a small proportion. Thus, there is a need to explore other different ways of personal cooling. Besides, some indexes for evaluating PCSs have been proposed, and they could be better and more universal if they could be used for evaluating different systems in terms of comfort and energy efficiency simultaneously. For example, the new index proposed by Schiavon and Melikov [13] is only for evaluating personal fans, while the Corrective Energy & Power index (CEP) proposed by He et al. [25] is only for personal electrical heating systems. More importantly, understanding the comments and the willingness of people with personal cooling will be pretty helpful for the development and application of personal cooling systems, but this point is usually ignored in previous studies. Some field investigations [26,27,28,29] showed that some people used personal cooling strategies (personal fans) to adapt to the ambient environment. However, few studies have really paid attention to understanding how people feel about personal cooling and what influences their willingness to adopt personal cooling.

The purposes of this study are listed below:

(1) To compare the comfort performances of several personal cooling systems, i.e., radiant cooling desk, desk fans and their combinations, in warm environments;
(2) To propose an improved *CEP* index which is possible to compare various personal cooling systems (not only those in this study);
(3) To understand how people feel about personal cooling and how they decide to adopt it.

To achieve these purposes, a series of experiments involving physical measurements, questionnaires and feedback was carried out in a real office environment. Then, with some results of our previous studies [23], three types of personal cooling systems (i.e., radiant cooling desk, desk fan and their combination) were compared. Afterwards, an improved *CEP* index was proposed to evaluate and compare various personal cooling systems. Besides, the comments of people who participated in the experiments were obtained and analyzed under two modes (i.e., they needed to pay for the personal cooling systems or not). Logistic regression was used to identify which factors influenced the willingness to adopt personal cooling.

## 2. Methods

### 2.1. Consent

Consent was obtained from all participants before the survey and the measurements. All involved people agreed to participate in the survey. It should be noted that this study mainly focused on the usage of personal cooling systems in a real office scenario, while no chemical, biological or medical tests were involved. All involved systems were widely used or studied in the real world and they did not cause any harm to people.

### 2.2. Location and Climate

This study was conducted in a building of Hunan University, Changsha, China (from June to August 2016 and June 2017). Changsha, a big city in the south-central China (111°53′–114°15′ E, 27°51′–28°41′ N), is situated in the Hot-Summer & Cold-Winter Zone in China. Changsha features a hot and humid climate in summer: the outdoor temperature often exceeds 30 °C while the relative humidity is close to 70% [30]. The outdoor temperature can be as high as 40 °C under extreme conditions. As for the humidity, it is hard to dehumidify indoor air in buildings even with air-conditioners in summer (the indoor relative humidity can reach higher than 70%) [27].

### 2.3. Experimental Room and Personal Cooling Systems

**Figure 1:**
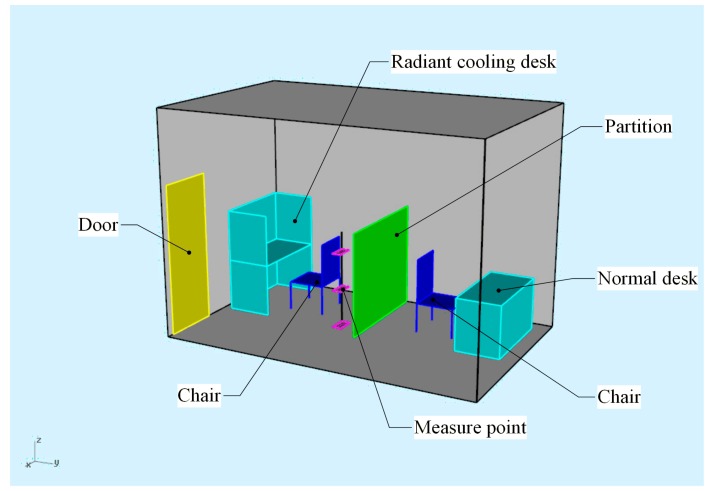
The office room for field experiments.

In order to create a realistic environment, all experiments were carried out in a real office room (length × width × height = 4.3 m × 2.7 m × 3.0 m) where several types of personal cooling systems were installed. As shown in Figure 1, two seats (one was radiant cooling desk and the other was a normal one) were prepared for the participants. A partition with thermal insulation layer and aluminum alloy surface was set up in the central of room. A detailed description of the room was presented in [23].

**Figure 2:**
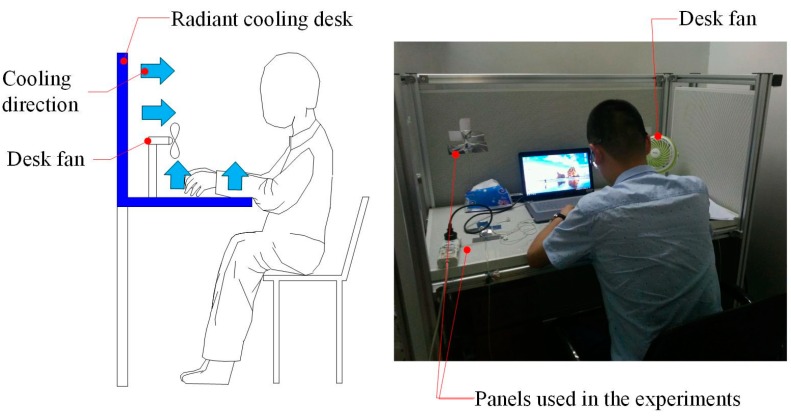
Radiant cooling desk and desk fan used in this study.

Figure 2 shows both the radiant cooling desk and the desk fan. The radiant cooling desk used water-cooled panels (1200 mm × 600 mm × 20 mm for each one). The panels had aluminum alloy surfaces and plastic capillary tubes insides. A rubber insulation layer (about 20 mm thick) was attached to the back of each panel so as to ensure the cooled surface was towards the users. Like the previous study [23], only two panels were used (marked in Figure 2). The two panels were supplied with cool water recirculated by a water chiller (only for experiments, not commercial air conditioning products). The temperature of cool water could be adjusted within the range from 10 to 24 °C. The size of desk fan was 180 mm × 180 mm × 270 mm (length × width × height). Two levels of local airflow speed (around 1.6 m/s and 2.2 m/s near the breathing zone of participants) could be produced by the desk fan, and the corresponding powers were 2 W and 3 W, respectively. The distance between the desk fan and participants was about 0.4 m. During the experiments, radiant cooling desk or/and desk fan were not controlled by the participants.

### 2.4. Experimental Procedure

**Figure 3:**
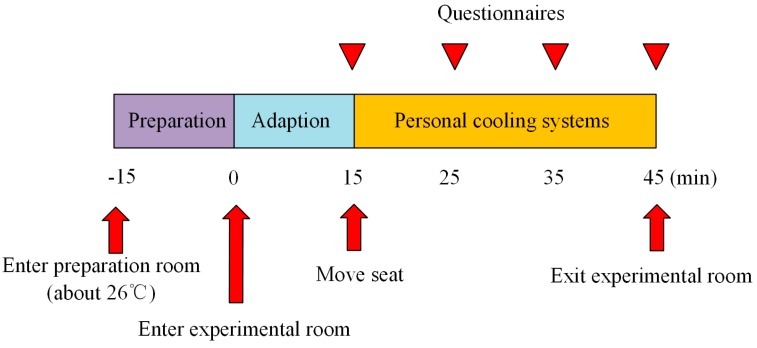
Experimental procedure.

As shown in Figure 3, the total time of each test was 1 h which consisted of three periods: 15 min in a preparation room (where the indoor temperature was around 26 °C), 15 min adaption at the normal seat and 30 min at the seat with radiant cooling desk and desk fan. The radiant cooling desk was running for about an hour prior to the test so as to reach stable surface temperature while the desk fan was turned on only when participants were seated near it. Participants did not drink alcohol or coffee, take medicine or stay up late before the experiments. During the tests, participants were offered a laptop so they could watch videos, surf the internet or listen to music. However, drinking, eating, smoking and walking were not allowed.

### 2.5. Experimental Conditions

In this study, the experiments mainly were conducted on the desk fan and the combination of radiant cooling desk and desk fan. Under the conditions with only the desk fan, the desk was not supplied with cool water. The indoor temperatures were set at 28 °C, 30 °C and 32 °C, respectively, corresponding to the summer climate in Changsha [30]. The relative humidity was kept about 60%. Details of the experimental conditions are presented in Table 1.

Based on the average values of physical parameters at the height of 0.6 m above the floor, the values of dew point, Predicted Mean Vote (PMV), Predicted Percentage of Dissatisfied (PPD) and Draught Risk were calculated or identified using the CBE Thermal Comfort Tool [31] (assuming clothing insulation 0.08 m^2^ K/W and metabolic rate 58.2 W/m^2^). Obviously, the environments in this study deviated from the comfort zone suggested by Standard ISO 7730 [32]. Also, the local air flow by desk fans was estimated to cause draught risk. However, the desk fan still improved thermal comfort in warm environments (see Section 3.1). The differences of supply and return water temperatures were kept within about 1 to 2 °C, which helped produce the uniform surface temperature for radiant panels. In addition, the average CO_2_ concentration in each test was usually lower than 500 ppm.

### 2.6. Participants

Twenty healthy university students (10 males and 10 females) who participated in our previous study [23] were invited to take part in this study again. All of them were born in the regions with hot climate in summer or had lived there for more than one year. All participants took part in each test of all conditions. The clothing insulation was about 0.5 clo (0.08 m^2^ K/W) during the experiments. The information of participants is shown in Table 2.

### 2.7. Instruments for Measurements

Measured physical parameters included air temperature (0.1 m, 0.6 m and 1.1 m), relative humidity (0.6 m), global temperature (0.6 m), air velocity (0.6 m), panel surface temperature, supply/return water temperature, water flow rate and so on. Details of instruments are listed in Table 1. According to the results of global temperature, air temperature and air velocity, mean radiant temperature (MRT) was calculated using CBE Thermal Comfort Tool [31] (results are shown in Table 3).

### 2.8. Questionnaires

The questionnaire included: (1) thermal sensation and comfort; (2) thermal acceptability and preference; (3) Air movement sensation and preference. Scales of subjective responses are presented in Table 4.

### 2.9. Cooling Capacity and the Improved CEP Index

The cooling capacity of radiant cooling desk was calculated as below:

(1) $$Q_{cool}=c\rho q\times \left(T_{out}-T_{in}\right)$$

where *Q_cool_* is the cooling capacity of radiant cooling desk (W), c is the specific heat capacity of water (4180 J/kg K), *ρ* is the density of water (1000 kg/m^3^), *q* is the water flow rate (m^3^/s), *T_out_* is the return water temperature (K) and *T_in_* is the supply water temperature (K).

The *CEP* index was firstly proposed in our previous study on personal heating systems [25]. It is defined as the energy efficiency of a certain type of PCSs when extending the acceptable temperature range. It is equal to the ratio between the energy consumed by PCS and its corresponding corrective power (*CP*). The energy refers to the electrical wattage of personal comfort systems in the steady-state condition. *CP* is a new index introduced by Zhang et al. [4] and it is defined as the difference between two ambient temperatures where people have the same thermal sensation: one without PCS and the other with PCS. What must be noted here is that *CP* is not the electrical wattage.

The value of *CEP* index is calculated as below:

(2) $$CEP=\frac{Q}{COP\times |CP|}$$

where *CEP* is the value of *CEP* index (W/K), *Q* is the average electrical power for each person with PCS (W) and |*CP*| is the absolute value of *CP* (K). Since the *CP* of personal cooling system is a negative value (<0) [4]. The lower *CP* indicates the larger ability to extend the acceptable temperature range, so the absolute value is used here. In this study, some changes were made to improve the *CEP* index:

(1) Coefficient of Performance (*COP*) was added in. Since water-cooling systems directly use cool water while fans consume electricity, *COP* is taken in for obtaining the equivalent electrical power. For water-cooling systems, *COP* can be determined by the water temperature, while for other cooling devices which used electricity (like fans), the *COP* value can be regarded as 1.0. As a result, it is possible to compare energy efficiencies of different personal cooling systems. As summarized in [33], when supply water temperature is higher than 16 °C, the *COP* of chiller can be higher than 8.5. Since the water temperature in this study was higher than 16 °C, the *COP* for radiant cooling desk was regarded as 8.5. The improved *CEP* index could be expressed as below:
  (3) $$CEP=\frac{Q^{\prime }}{COP\times |CP|}$$
  where *Q*’ is the average cooling energy (W). For water-cooling systems, it is the value of average cooling capacity. For fans, it is the value of average electrical power.
  When multiple personal cooling systems are used simultaneously, the *CEP* value is equal to the sum of those of all systems. Subsequently, the *CEP* value can be calculated as below:
  (4) $$CEP=\overset{N}{\underset{n=1}{\sum }}\frac{{Q^{\prime }}_{n}}{COP_{n}\times |CP_{n}|}$$
  where *n* is the serial number of personal cooling systems, *N* is the total number of personal cooling systems used simultaneously under one condition.
(2) Different comfort levels were defined. Corresponding to 80% and 90% acceptable range, thermal sensation votes are within the range of −0.85 to +0.85 and −0.5 to +0.5, respectively [16]. Thus, according to the results of thermal sensation votes corresponding to 80% and 90% acceptable range, respectively, *CEP* were calculated and expressed in two ways: *CEP*_80%_ and *CEP*_90%_. Further, similar with [25], the average value of *CEP* and the lowest *CP* (meaning the largest ability to extend the acceptable temperature range) can be used together as a simplified way to present the performances of personal cooling systems. Thus the simplified *CEP* index is expressed as ${\bar{CEP}}_{80\%}\left(CP_{max}\right)$ or ${\bar{CEP}}_{90\%}\left(CP_{max}\right)$ (${\bar{CEP}}_{80\%}$ and ${\bar{CEP}}_{90\%}$ are average values of *CEP* values when thermal sensation votes are within 80% and 90% acceptable ranges, respectively).
(3) A common baseline (without personal cooling systems) was defined for comparing different personal cooling systems. Thus, *CP* values of different personal cooling systems could be comparable. The common baseline is set at the temperature of 26 °C. Please refer to Section 3.2 for details.

### 2.10. The Feedback

After the experiments, all people were invited to complete another special task called the *Feedback*. This required participants to: (1) report any discomfort symptoms they had during the experiments and (2) provide comments on the personal cooling systems. In the latter part, the comments could be divided into two categories: (1) the opinion and (2) the willingness. The opinions were mainly related to four factors: the easiness, the effectiveness, the economy and the safety, corresponding to those in our previous field investigation on personal heating devices [34]. Also, the price of three types personal cooling systems were given, i.e., 1600 RMB (about 240 US dollars) for radiant cooling desk, 40 RMB (about 6 US dollars) for desk fan and 1640 RMB (about 246 dollars) for their combination. The willingness to adopt a certain type of personal cooling systems was inquired under two circumstances: participants had to pay or not (i.e., charge mode and free mode). Since all participants gave comments on three types of personal cooling respectively, there were 60 sets of comments in total. Moreover, if participants answered “No” to the questions related to the willingness, they were asked to give reasons. The questions related to the comments are listed in Table 5.

### 2.11. Statistical Analysis

All statistical analyses were performed by IBM SPSS Statistics software (SPSS Inc., Chicago, IL, USA). In order to better identify the performances of different personal cooling systems, the results of conditions with radiant cooling desk and without personal cooling from [23] were also presented as references. Corresponding to the experimental conditions in this study, at the certain air temperature, the main variable was the personal cooling strategy (the two levels of local airflow by fans were regarded as different cooling strategies). Under this circumstance, the obtained results were firstly tested by Kolmogorov-Smirnov test to identify the normality. Then, normally distributed data were tested by paired *t*-test, while the others by non-parametric Friedman test. Statistical differences were regarded as significant at the 0.05 level (i.e., *p* < 0.05).

Besides, in order to understand the factors influencing people’s willingness to adopt personal cooling systems, logistic regression was used to analyze the relationship between the opinions (easiness, effectiveness, economy and safety) and the willingness (whether use or not) towards different personal cooling systems. The positive answers (“Yes”) were set as 1, while the negative (“No”) ones as 0. The four types of opinions were regarded as the independent variables and the willingness were regarded as the dependent ones. Firstly, four opinions were all inputted and the software began the first regression. Then, the software gave the results of pre-regression significances of all variables. In order to improve the precision of regression model, the insignificant variables (*p* > 0.05) were excluded. Afterwards, the significant variables were inputted and the software began the second regression. Lastly, the logistic regression was obtained. The logistic regression model is expressed as below:

(5) $$\mathrm{logit}\left(p\right)=\beta_{0}+\overset{N}{\underset{n=1}{\sum }}\beta_{n}x_{n}$$

where *p* is the probability of adopting personal cooling systems, *x_n_* is the significant independent variables, *N* is the number of significant independent variables) and *β*_0_ and *β_n_* (*n* = 1 to *N*) are the regression coefficients.

## 3. Results

### 3.1. Comfort Performances of Personal Cooling Systems

#### 3.1.1. Thermal Sensation and Comfort

**Figure 4:**
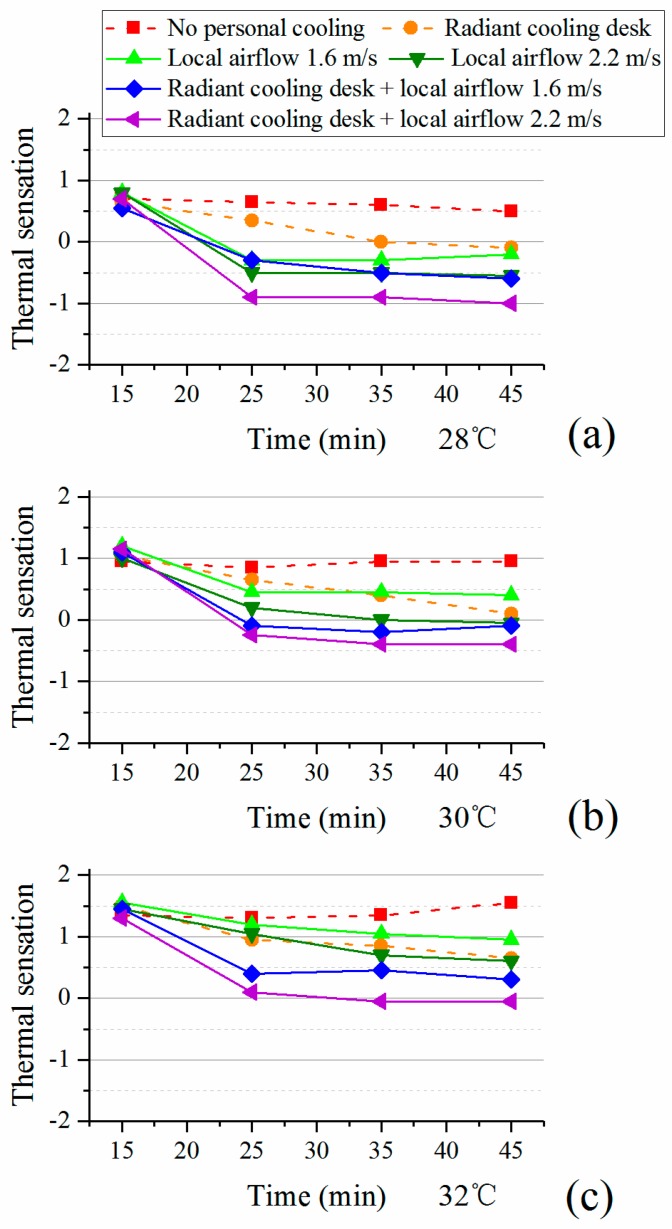
Thermal sensation with personal cooling systems: (**a**) 28 °C; (**b**) 30 °C; (**c**) 32 °C.

Figure 4 illustrates the average votes of thermal sensation under different conditions (the results in [23] are presented for reference by using dashed lines). As compared to the conditions without personal cooling, all personal cooling systems reduced the warmth sensation after participants used them for 10 min and the thermal sensation vote remained relatively stable during the last 20 min. Among all conditions, the combination of radiant cooling desk and desk fan always exerted the greatest cooling effect on participants since the corresponding thermal sensation votes were the lowest ones in the environment at a certain temperature. The desk fan also rapidly cooled the participants within 15 min. As for radiant cooling desk, it took more time to reach a similar sensation level.

In order to further explore the comfort differences among different personal cooling systems, the last votes in each test were regarded as those in the steady-state condition and were used for comparisons. Table 6 presents the comfort differences. Along with results in Figure 4, it can be seen that all personal cooling strategies made participants feel significantly cooler. Meanwhile, the combination of radiant cooling desk and desk fan often achieved significantly lower thermal sensation votes than using radiant cooling desk or desk fan alone.

**Figure 5:**
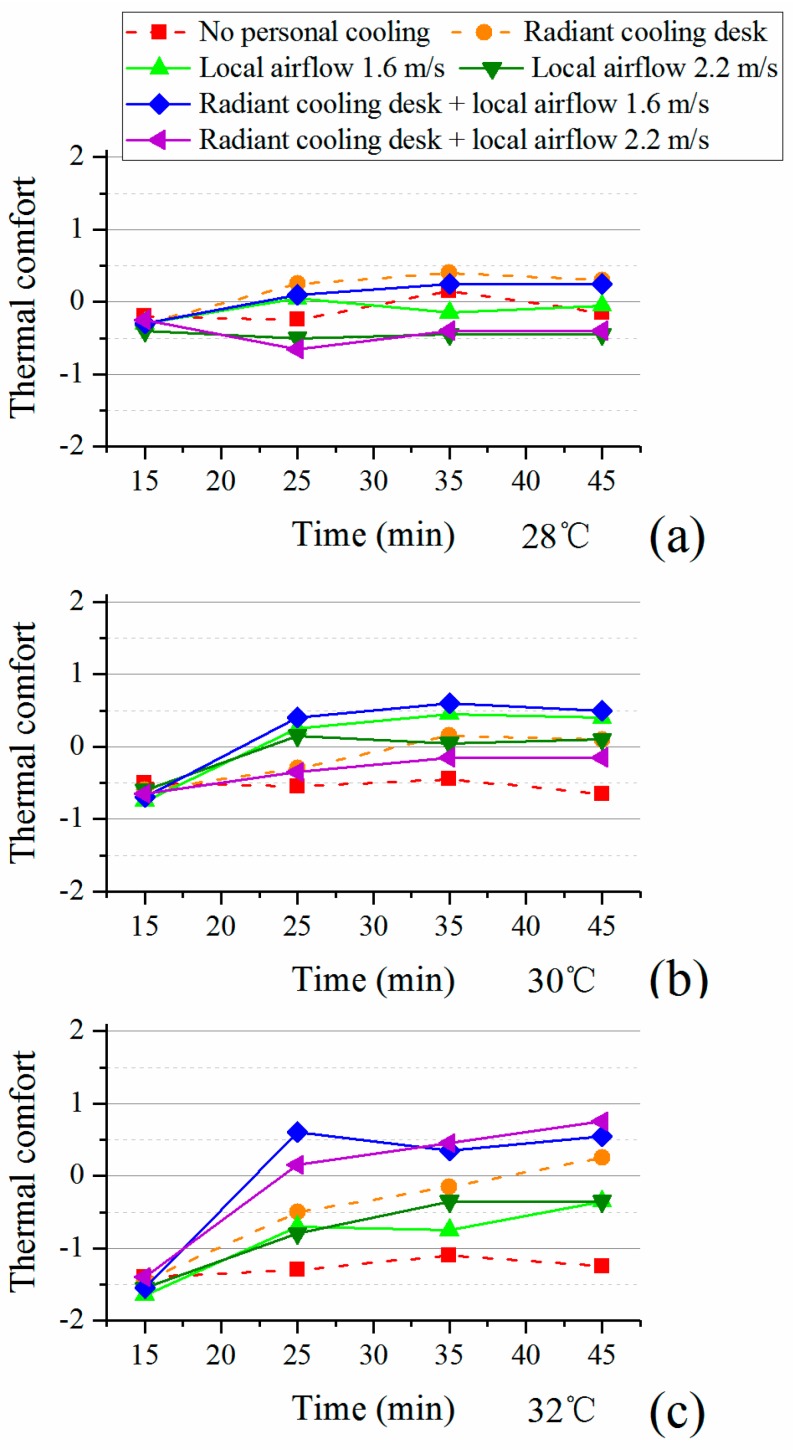
Thermal comfort with personal cooling systems: (**a**) 28 °C; (**b**) 30 °C; (**c**) 32 °C.

The average votes of thermal comfort were shown in Figure 5. Corresponding to the results of thermal sensation (Figure 4), the obvious changes of thermal comfort happened from the 15th to the 25th minute and then participants were at a relatively stable comfort level. However, different from the results of thermal sensation, personal cooling showed complex effects on thermal comfort. At 28 °C, personal cooling could ever cause lower comfort level due to the over-cooling (see Figure 4).

As air temperature increased to 30 °C and 32 °C, all personal cooling systems exerted positive effects on comfort. The combination achieved the highest comfort level at both 30 °C and 32 °C. Although it was estimated that the desk fan would cause the draught, it produced similar comfort level as radiant cooling desk and the combination did under the condition at 30 °C. Nonetheless, its effect was weakened as indoor air temperature went up to 32 °C. This indicates that the actual draught risk in hot environments is lower than that predicted by the current standard.

Table 7 shows the results of comfort differences. The comfort differences were enlarged as indoor air temperature increased. Whatever the personal cooling strategy was, it achieved significantly better comfort under the conditions at 30 °C and 32 °C than the conditions without. Specially, participants with the combination of radiant cooling desk and desk fan had significantly higher comfort level than other personal cooling strategies. Thus, radiant cooling desk was more suitable at 28 °C while the combination had the best comfort performance at 30 °C and 32 °C.

#### 3.1.2. Thermal Acceptability and Preference

**Figure 6:**
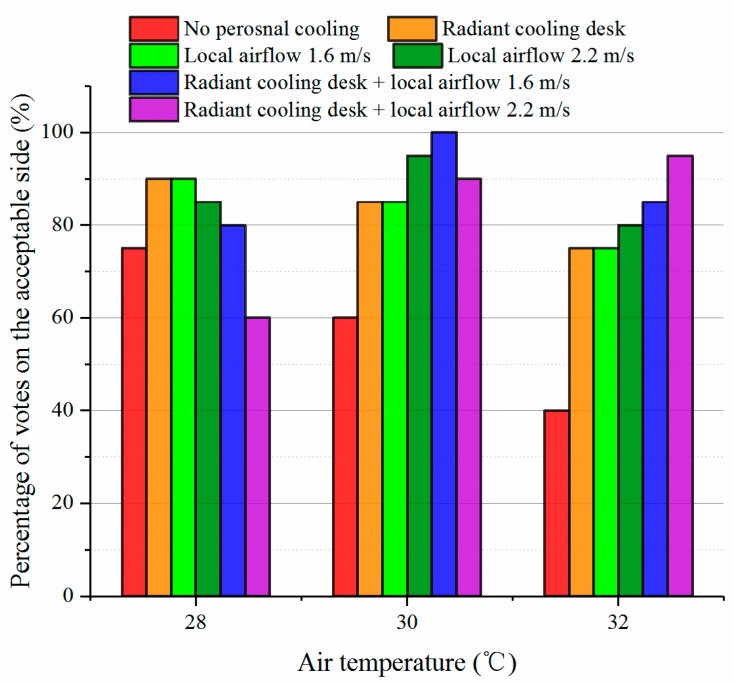
Thermal acceptability votes on the acceptable side.

In this study, participants who voted “Slightly acceptable”, “Acceptable” or “Totally acceptable”) were regarded as those on the acceptable side, while the others on the unacceptable side. Figure 6 displays the percentages of participants on the acceptable side in the last votes under different conditions. It is clear that personal cooling systems improved thermal acceptability under most of conditions. Besides, corresponding to the comfort results in Figure 5, using single personal cooling system (radiant cooling desk or desk fan) had the highest percentage of acceptable under the condition where the temperature was 28 °C, while the combination made more than 90% of participants vote on the acceptable side as the indoor temperature rose to 30 °C and 32 °C. The low acceptable percentage with the combination under the condition at 28 °C can be traced back to the cool sensation (see Figure 4).

**Figure 7:**
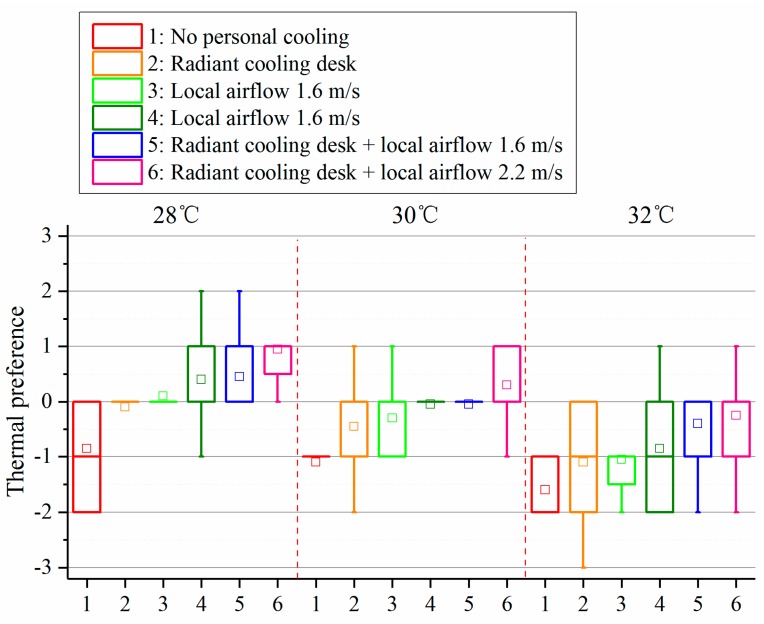
Thermal preference votes in the last votes.

Figure 7 shows the results of thermal preference in last votes under different conditions. The votes of “Slightly cooler”, “Cooler” and “Much cooler” were regarded as those on the cooler side, while “Slightly warmer”, “Warmer” and “Much warmer” on the warmer side. Under the condition at 28 °C, radiant cooling desk or local airflow at 1.6 m/s made most participants vote on the neutral level, while other personal cooling strategies made at least 40% participants prefer to be warmer. As the environment became warmer, preference votes shifted to the cooler side, while combining radiant cooling desk and desk fan kept more than half of participants preferring “No change”.

#### 3.1.3. Air Movement Sensation and Preference

**Figure 8:**
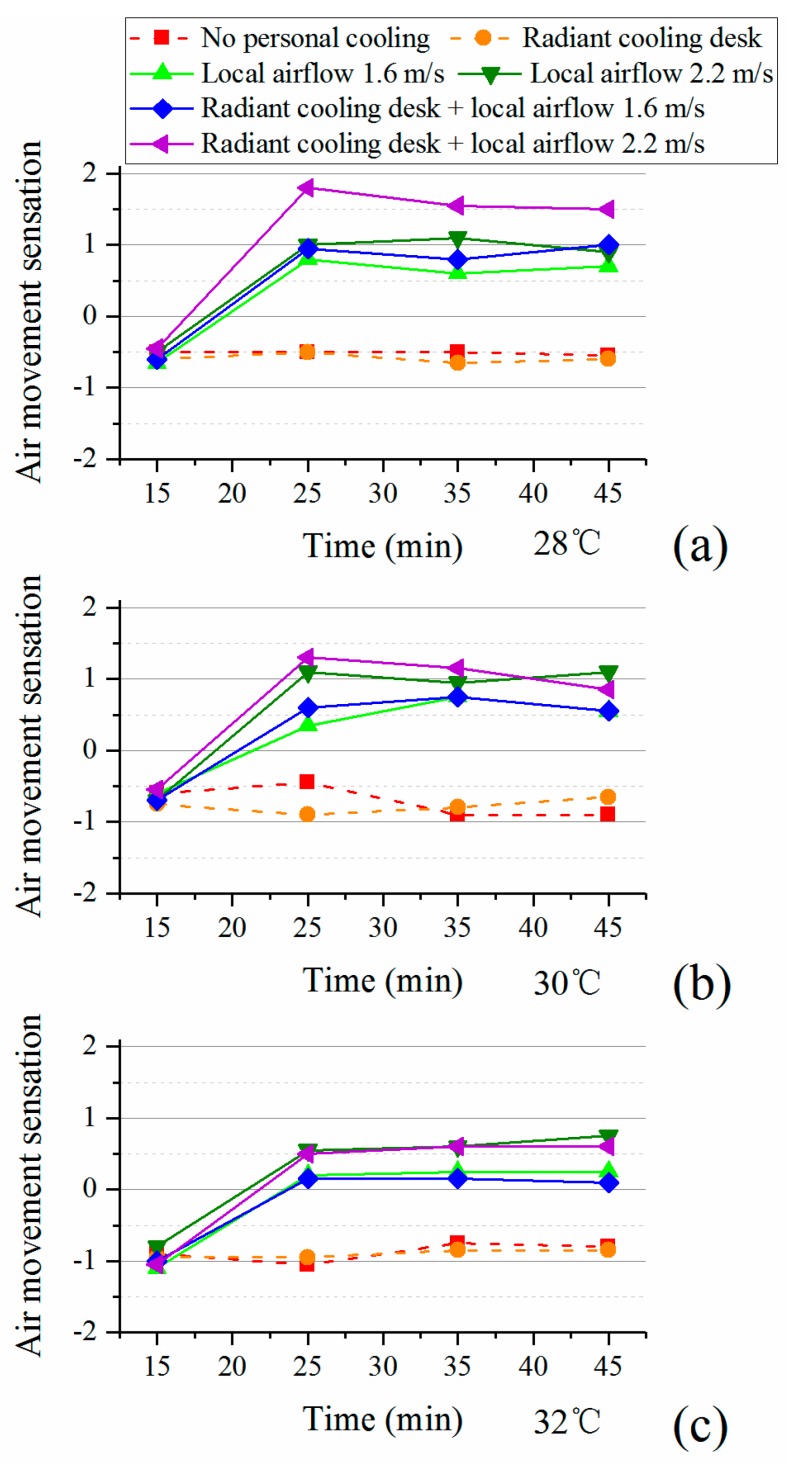
Air movement sensation with personal cooling systems: (**a**) 28 °C; (**b**) 30 °C; (**c**) 32 °C.

Figure 8 illustrates the changes of air movement sensation during the experiments. Whatever the indoor temperature was, an obvious increase of air movement sensation was observed as participants used the desk fan for 10 min, while the sensation remained stable for the rest of the time. Also, at three levels of air temperature, the use of desk fan caused significant differences (*p* < 0.05) of air movement sensation as compared to conditions without personal cooling or with merely radiant cooling desk. Another phenomenon worth noting is that participants with the desk fan had weaker air movement sensation when exposed to the warmer environment. This indicates that participants accepted a strong local airflow as the indoor temperature increased.

The results of air movement preference in the last votes are presented in Table 8. As the environment became warmer, participants preferred stronger air movement whether they were with or without personal cooling. Each type of personal cooling system reduced the preference for stronger air movement, specially the desk fan (whether with or without radiant cooling desk). Moreover, with the combination of radiant cooling desk and desk fan, the average votes of air movement preference were always on the weaker side and nearly no participants preferred stronger air movement.

#### 3.1.4. Other Discomfort Symptoms

The results of discomfort symptoms obtained from the feedback were summarized and presented in Table 9. It is obvious that participants complained mostly about the stuffy air indoors during the experiments although the CO_2_ concentration was not high (see Section 2.5). With the help of personal cooling, the complaints about the stuffy air were reduced. Specially, the use of a desk fan greatly ameliorated this discomfort. Meanwhile, the use of the desk fan lowered the frequency of complaints about the humidity. Nonetheless, dizzy feelings and dry eyes became obvious with the desk fan. Also, some participants who felt dizzy clearly attributed these two symptoms to it. Moreover, some participants felt fatigue. One possible reason for this was that the experiments were conducted at the end of semester when students were busy preparing for final examinations. In addition, although some other discomfort symptoms appeared during the experiments, like dry skin, itchy skin, dry lips and thirsty, their frequencies seldom exceeded 10%.

### 3.2. Energy Efficiencies and CEP Index

**Figure 9:**
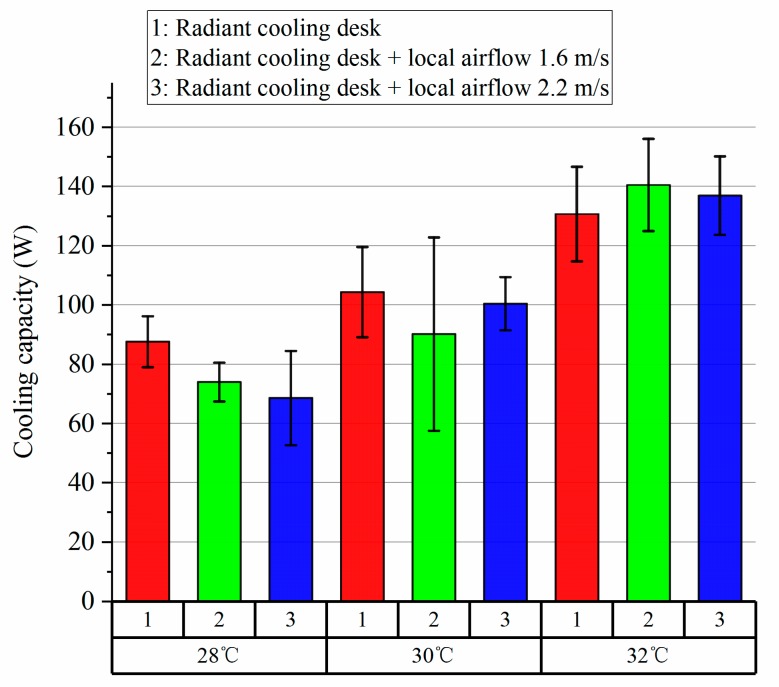
Cooling capacity of radiant cooling desk under different conditions.

For the cooling capacity of radiant cooling desk, it was firstly calculated at each minute and then its average value and standard deviation was figured out. Figure 9 presents the results of the cooling capacity of the radiant cooling desk. The cooling capacity of the radiant cooling desk was elevated as the indoor temperature was higher. The deviation of cooling capacity mainly resulted from the variation of supply/return water temperatures for maintaining the relatively stable temperature of the panel surfaces.

**Figure 10:**
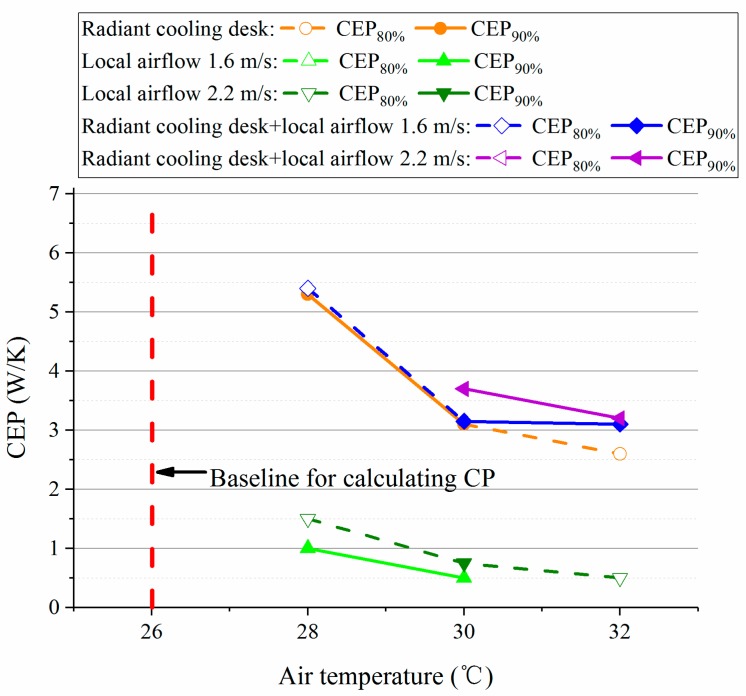
The calculated *CEP* values of personal cooling systems in this study.

In this study, since the air temperature in the preparation room was 26 °C where the corresponding PMV value was very close to the neutral level (calculated by the CBE Thermal Comfort Tool [31]), 26 °C was regarded as the baseline for calculating *CP* in this study. Then, according to the method in Section 2.9, the *CEP* values were calculated and presented in Figure 10. 

It can been see that the desk fan was superior to the other personal cooling strategies in terms of energy efficiency. Nonetheless, its effect on extending the acceptable temperature range was limited. On the contrary, the combination of radiant cooling desk and desk fan consumed more energy while it achieved better comfort in the environment at 32 °C. However, it may overcool the participants (see Section 3.1.1) so it was not as good as other two systems under the condition at 28 °C. Moreover, it seems that the radiant cooling desk was a middle choice because it well maintained neutral thermal sensation at 28 °C and 30 °C and its energy-efficiency was higher than the combination at 32 °C. The analyses on different personal cooling strategies were further extended in Section 4.1.

### 3.3. Comments on Personal Cooling Systems and the Adoption Model

**Figure 11:**
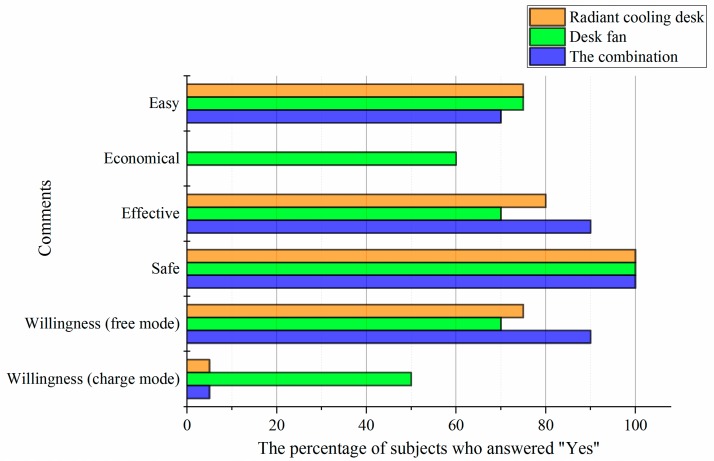
Percentages of participants who gave positive comments on personal cooling systems.

As shown in Figure 11, participants gave different comments on personal cooling systems. More than 70% of participants considered three types of personal cooling systems were easy to use, while only 60% thought the desk fan was economical and no person thought so for the radiant cooling desk and the combination. Besides, corresponding to the results in Section 3.1.1, the radiant cooling desk and the combination made more participants feel that their warmth sensations were effectively reduced. As for safety, all participants considered personal cooling was safe. Moreover, when it came to the willingness to adopt personal cooling systems, a large difference was observed between the free and the charge modes. Although personal cooling systems were effective, only one person was willing to buy the radiant cooling desk or the combination, while only half of participants reported that they’d like to pay for the desk fan although it’s not expensive. Nevertheless, if personal cooling was free, the percentage of participants who’d like to adopt them sharply increased to 75% or higher.

**Figure 12:**
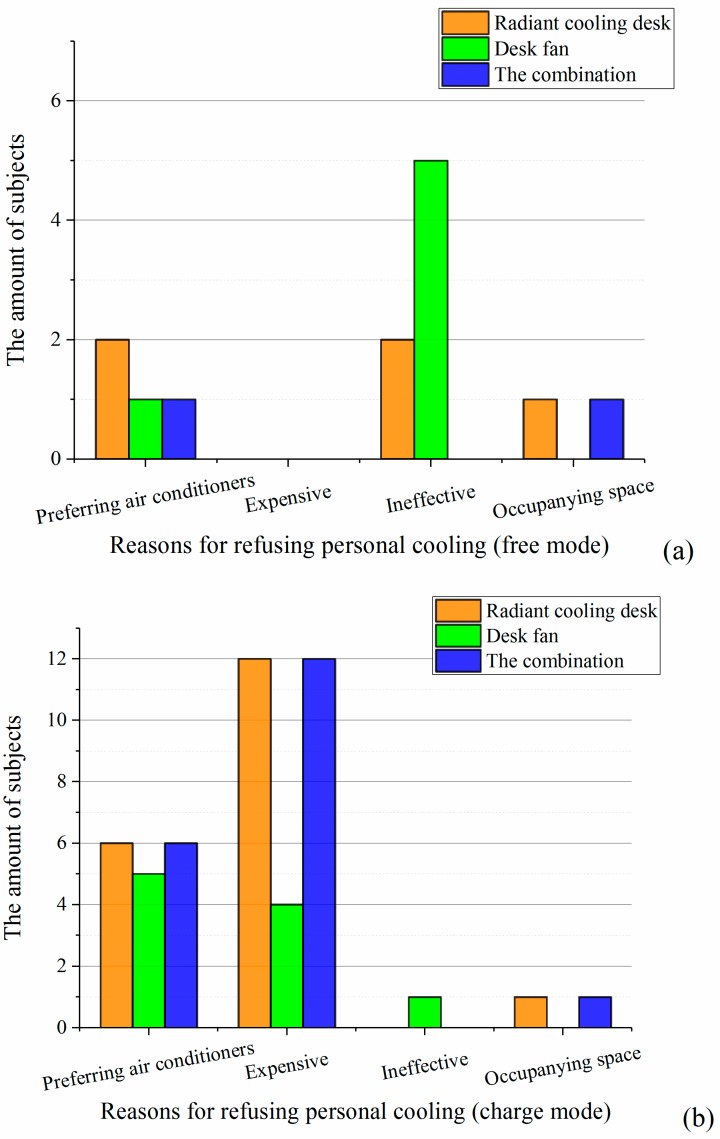
Reasons for refusing to adopt personal cooling systems: (**a**) free mode; (**b**) charge mode.

Figure 12 presents reasons why participants refused to adopt personal cooling. “Preferring air conditioners” means participants preferred conventional air conditioners or they thought air conditioners were enough in their daily life so they did not need personal cooling. Under the free mode, the largest reason for refusing was the ineffectiveness of personal cooling, followed by the one that participants preferred air conditioners. When it comes to the charge mode, most of participants refused personal cooling due to it cost. Also, some participants thought if they had to pay for personal cooling, they’d like to choose conventional air conditioners instead. In addition, whatever the mode was, one person still considered the radiant cooling desk and the combination occupied extra space which was reason for refusing. In this study, preferring conventional air conditioners was an unexpected reason for refusing personal cooling. This question was further discussed in Section 4.2.

Table 10 presents the logistic regression results. Under the free mode, the effectiveness was the only significant factor that affected the adoption of personal cooling systems. The positive value of β indicates that effective personal cooling systems would lead to a greater possibility of adoption. This result is consistent with that in Figure 12 that participants was unwilling to adopt ineffective personal cooling systems although they were free. However, under the charge mode, only the economy exerted the significant effect on the adoption. Meanwhile, economical personal cooling systems would increase the willingness of participants to pay for them (the value of β was positive). Besides, this result was also consistent with that in Figure 12 that the main obstruction for adopting non-free personal cooling was the cost.

## 4. Discussion

### 4.1. Comparisons among Different Personal Cooling Systems

Firstly, three types of personal cooling strategies in this study were compared. The results of the improved *CEP* index in Section 3.2 indicates that the hybrid one (i.e., the combination of radiant cooling desk and desk fan) had the advantage of widening the comfortable temperature range, while the single personal cooling (radiant cooling desk or desk fan) had the higher energy-efficiency. Nonetheless, there were some other factors influencing subjective comfort. When the desk fan was in use (whether combined with radiant cooling desk or not), fewer participants complained about the humid environment and the stuffy air (see Section 3.1.4). This is consistent with some previous studies that increased air velocity could lower the humid sensation [21,35] and improve the perceived air quality [17,36,37]. However, the strengthened airflow caused dizzy feelings and dry eyes in this study. Also, participants with the desk fan were faced with a draft risk and they preferred weaker airflow (see Section 3.1.3). As for radiant cooling desk, although it avoided the draft risk and dry-eye discomfort, it was not so effective at fully eliminating the complaints about humidity and indoor air as the desk fan did. Since participants could not adjust personal cooling systems in this study, maybe allowing participants to control the combination of radiant cooling desk and desk fan according to their actual requirements will be a better solution for trading off different personal cooling strategies.

**Figure 13:**
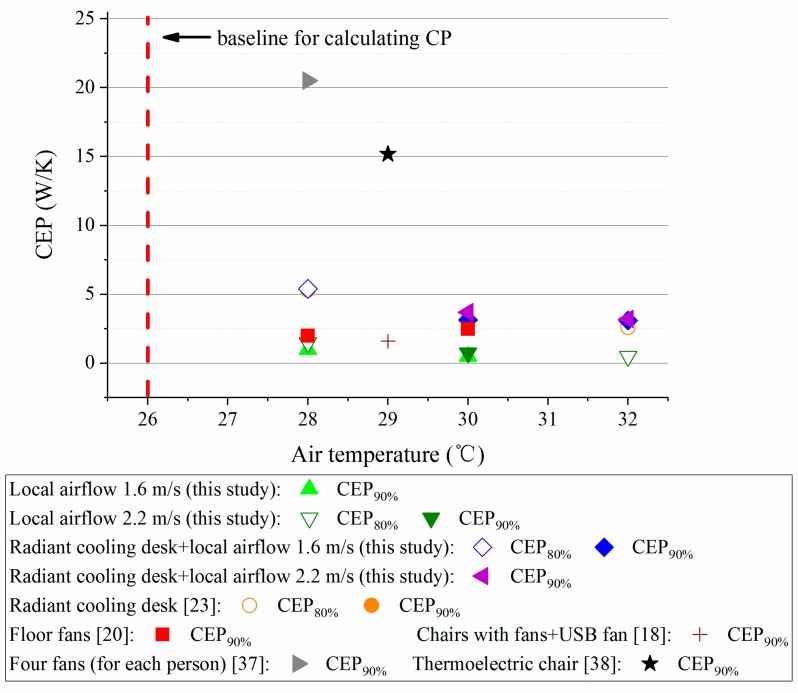
Comparisons with previous studies on personal cooling systems.

In order to further clarify the performances of the combination of radiant cooling desk and desk fan, the comparisons with some typical previous studies on personal cooling [18,20,37,38] were conducted by using the improved *CEP* index. Mostly, the previous studies were conducted by the research group at University of California, Berkeley. Table 11 lists the basic information of personal cooling systems as well as the simplified *CEP* values, while Figure 13 illustrates the detailed results of *CEP* values under different conditions. Similar to the results in Section 3.2, personal fans exerted good comfort performance when the ambient temperature was not higher than 30 °C. Also, personal fans could achieve high energy-efficiency than other personal cooling systems. Radiant cooling desk or the combination needed more energy but kept better comfort when the ambient air temperature was higher than 30 °C. An important reason for the difference goes to the fact that personal fans since they merely recirculate the indoor air and the micro-environment near body parts is not cooled (air temperature remains unchanged). Thus personal fans could not fully eliminate warm sensation when the indoor air temperature was reached 30 °C or higher [21,22,39]. As for the radiant cooling desk [23] and the thermoelectric chair [38], they directly absorbed heat from human bodies, a feature which produced a cool micro-environment for the participants under the hot conditions.

Moreover, according to the study of Hoyt et al. [7], the energy consumption of air conditioning system in buildings can be lowered by around 7% as the indoor set point temperature is 1 °C higher in summer. As a result, applying various personal cooling systems simultaneously could save more energy of the central air conditioning system. Thus, although the combination of radiant cooling desk and desk fan needed more electricity, more energy of the central air conditioning system) could still be saved than using personal fans alone. This issue needs further studies in future.

### 4.2. Adoption of Personal Cooling Systems

As discussed in Section 4.1, personal cooling systems contribute to extending the comfortable temperature range and saving energy. However, in the real world, personal cooling is not as popular as conventional air conditioning systems. Zhang et al. [4] claimed that the main reasons for the lack of the adoption of personal cooling systems were: (1) the separate cost and benefit (the users pay for personal cooling systems while the energy-efficiency benefit belongs to the landlord) and (2) the complex connection to the central system. This study presents some different reasons.

Under the free mode, participants chose the system that was effective to reduce warm sensation, while the economy factor was insignificant. This is because people will primarily choose to be comfortable if people don’t have to pay for the corresponding measures [40]. Thus, whether expensive or cheap, effective personal cooling owns the superiority.

The charge mode is close to the actual condition in residential buildings. Under this circumstance, the most important factor was the economy. This is consistent with the opinion of [41,42] that the low cost is a driving factor to adopt energy-saving measures. However, the effectiveness was not a significant factor here. Direct evidence supporting this result is presented in [42]: in residential buildings, occupants’ concern about the cost plays a more important role than the requirement for comfort. Moreover, another essential reason for refusing personal cooling was related to conventional air conditioners. This is due to the fact that there had already been air conditioners in the buildings where the participants often stayed (like classrooms, offices and dormitory rooms). Thus, there was no need for them to buy or use extra personal cooling systems. In other words, the existence of conventional air conditioners will lower the willingness of people to adopt personal cooling. This point was supported by our previous field investigation in air-conditioned dormitory buildings [27] where nearly half of participants reported that they may use fans in summer, but actually few of them were really using them during the investigation. Moreover, another evidence is presented in a field study conducted in Japan [28]: the frequency of switching on stand fans was lower than 6% in the room with air conditioners, while higher than 19% in the room without. Considering the factors that hinder the adoption of personal cooling systems, free and effective personal cooling systems should be regarded as a better choice to achieve the wider application.

### 4.3. Limitations and Suggestions

Some limitations of this study are clarified here which will be helpful for the studies in future. Firstly, this study focused on the performance of personal cooling systems. The building cooling load with personal cooling systems was not involved. Probably, it is different from that with conventional air conditioning systems. Also, the improved *CEP* index provided an easy way to compare different personal cooling systems in terms of comfort and energy-efficiency. However, this index is mainly based on thermal sensation rather than the actual comfort because the latter one is very complex and is influenced by various other factors not related to the thermal environment or the personal cooling system. Future studies should distinguish the effect of personal cooling on comfort from those of other factors.

What’s more, the radiant cooling desk was an experimental system rather than commercial product, and its cost was still high. Besides, as shown in Section 3.1.1, the combination of radiant cooling desk and desk fan overcooled the participants under the condition at 28 °C, which means the optimization for better comfort is needed.

Lastly, the logistic regression models were established by using the comments of university students. Although some useful information was obtained, there is still the need to understand the comments of different people.

## 5. Conclusions

Main conclusions are summarized as below:

(1) Personal cooling systems reduced the warmth sensation and improved the comfort of participants in warm environments. Specially, the combination of radiant cooling desk and desk fan well extended the acceptable temperature range to 32 °C.
(2) The radiant cooling desk lowered the draft risk and the desk fan reduced participants’ complaints about the humid environment and the indoor air. Thus allowing personal control over their combination maybe a better choice to further improve subjective comfort.
(3) The improved *CEP* index was proposed and it provided a practical way to compare different types of personal cooling systems in terms of comfort and energy efficiency at the same time.
(4) According to the results of our improved *CEP* index, desk fans were more energy-efficient and suitable for participants in the environment where the air temperature was not higher than 30 °C, while the combination of radiant cooling desk and desk fan consumed more energy, but was superior to desk fans in terms of extending the comfortable temperature range.
(5) Under the free mode, most of participants were willing to adopt personal cooling systems and the effectiveness was the main factor influencing the willingness, while under the charge mode, most participants did not want to adopt personal cooling due to its perceived cost and the availability of conventional air conditioners.

## Figures and Tables

**Table 1:** Experimental conditions.

Physical Parameters ^1^	Conditions					
	1	2	3	4	5	6
Radiant cooling desk	No	No	No	No	No	No
Local airflow ^2^ (m/s)	1.6	2.2	1.6	2.2	1.6	2.2
Air temperature 1.1 m (°C)	28.5 ± 0.5	28.7 ± 0.4	30.8 ± 0.4	30.7 ± 0.4	33.0 ± 0.4	32.8 ± 0.5
Air temperature 0.6 m (°C)	27.8 ± 0.5	28.1 ± 0.5	30.2 ± 0.4	29.8 ± 0.5	32.3 ± 0.5	32.0 ± 0.4
Air temperature 0.1 m (°C)	27.1 ± 0.3	27.2 ± 0.4	29.0 ± 0.4	28.7 ± 0.5	31.6 ± 0.4	31.3 ± 0.3
Relative humidity (%)	57 ± 5	60 ± 6	62 ± 4	59 ± 5	59 ± 4	63 ± 4
MRT (°C)	28.0 ± 0.4	28.3 ± 0.4	30.7 ± 0.3	30.2 ± 0.3	32.5 ± 0.3	32.3 ± 0.4
Air velocity (m/s)	0.06 ± 0.03	0.05 ± 0.04	0.09 ± 0.03	0.07 ± 0.03	0.05 ± 0.04	0.06 ± 0.03
Dew point (°C)	-	-	-	-	-	-
PMV	0.83	0.97	1.80	1.65	2.57	2.52
PPD	19%	25%	67%	59%	95%	94%
	7	8	9	10	11	12
Radiant cooling desk	Yes	Yes	Yes	Yes	Yes	Yes
Local airflow ^2^ (m/s)	1.6	2.2	1.6	2.2	1.6	2.2
Air temperature 1.1 m (°C)	28.8 ± 0.5	28.6 ± 0.5	31.0 ± 0.4	30.7 ± 0.3	32.9 ± 0.4	32.6 ± 0.4
Air temperature 0.6 m (°C)	28.2 ± 0.4	27.9 ± 0.5	30.3 ± 0.4	29.9 ± 0.4	32.2 ± 0.3	32.0 ± 0.4
Air temperature 0.1 m (°C)	26.8 ± 0.4	26.8 ± 0.4	29.3 ± 0.3	28.9 ± 0.4	31.4 ± 0.3	31.1 ± 0.3
Relative humidity (%)	63 ± 3	61 ± 4	58 ± 4	64 ± 5	62 ± 3	59 ± 4
MRT (°C)	28.5 ± 0.3	28.4 ± 0.3	30.6 ± 0.3	30.2 ± 0.2	32.3 ± 0.2	32.3 ± 0.3
Air velocity (m/s)	0.08 ± 0.03	0.07 ± 0.02	0.07 ± 0.02	0.08 ± 0.03	0.06 ± 0.03	0.07 ± 0.02
Dew point (°C)	20.4	19.5	21	22.2	23.9	22.9
Supply water (°C)	19.7 ± 1.3	19.5 ± 1.3	18.5 ± 1.8	18.6 ± 2.1	17.6 ± 2.2	17.4 ± 2.6
Return water (°C)	20.9 ± 1.0	20.4 ± 1.1	19.6 ± 1.2	19.9 ± 1.5	19.4 ± 1.7	19.2 ± 2.2
Panel surface (°C)	23.3 ± 1.0	23.4 ± 1.2	24.1 ± 1.5	24.2 ± 1.2	24.7 ± 1.3	24.3 ± 1.4
PMV	1.04	0.96	1.80	1.70	2.54	2.46
PPD	28%	24%	67%	61%	94%	92%

^1^ Average value ± Standard deviation; ^2^ Airflow by desk fans in breath zone (near the faces of participants), measured by pre-tests.

**Table 2:** Information of participants.

Participants	Sample Size	Age	Height (cm)	Weight (kg)	Body Mass Index (kg/m^2^)
Female	10	23.5 ± 1.0	163.5 ± 3.9	49.5 ± 4.0	18.5 ± 1.3
Male	10	24.1 ± 1.6	169.9 ± 3.4	63.8 ± 9.6	22.1 ± 3.4
All	20	23.8 ± 1.3	166.7 ± 4.7	56.7 ± 10.0	20.3 ± 3.1

**Table 3:** Measurement instruments.

Parameters	Instruments	Accuracy
Air temperature Relative humidity	TR-72Ui temperature and humidity meter	±0.3 °C
		±5%
Global temperature	TR-102 black globe temperature meter	±0.2 °C
Air velocity	VELOCICALC-8347 air velocity meter	±3%
CO2 concentration	TSI-8762 indoor air quality meter	±3%
Water flow rate	LWGYS-C flow meter	±1%
Water temperature	Pt100 thermometer	±0.15 °C
Panel surface temperature	Pt100 thermometer (surface mount type)	±0.15 °C

**Table 4:** Scales of subjective responses.

Scale	Thermal Sensation	Thermal Comfort	Thermal Preference	Air Movement Sensation	Air Movement Preference	Scale	Thermal Acceptability
3	Hot	Very comfortable	Much warmer	Very strong	Much stronger	6	Totally acceptable
2	Warm	Comfortable	Warmer	Strong	Stronger	5	Acceptable
1	Slightly warm	Slightly comfortable	Slightly warmer	Slightly strong	Slightly stronger	4	Slightly acceptable
0	Neutral	No feeling	No change	Neutral	No change	3	Slightly unacceptable
−1	Slightly cool	Slightly uncomfortable	Slightly Cooler	Slightly weak	Slightly weaker	2	Unacceptable
−2	Cool	Uncomfortable	Cooler	Weak	Weaker	1	Totally unacceptable
−3	Cold	Very uncomfortable	Much cooler	Very weak	Much weaker		

**Table 5:** Questions related to the comments on personal cooling systems in the Feedback.

Categories	Factors	Mode	Questions
Opinion	Easiness	-	Do you think this system is easy to use?
Opinion	Effectiveness	-	Do you think this system is effective to reduce warm sensation?
Opinion	Economy	-	Do you think this system is economical?
Opinion	Safety	-	Do you think this system is safe?
Willingness	-	Free	Will you adopt this system if it is totally free?
Willingness	-	Charge	Will you adopt this system if you have to pay for it?

**Table 6:** Thermal sensation differences among personal cooling systems.

Conditions	Personal Cooling Strategies			
Radiant cooling desk	No	No	Yes	Yes
Local airflow 2 m/s	1.6	2.2	1.6	2.2
28 °C				
No personal cooling	*	*	*	*
Radiant cooling desk	None	None	*	*
Local airflow 1.6 m/s	-	None	None	*
Local airflow 2.2 m/s	-	-	None	*
Radiant cooling desk + local airflow 1.6 m/s	-	-	-	*
Radiant cooling desk + local airflow 2.2 m/s	-	-	-	-
30 °C				
No personal cooling	*	*	*	*
Radiant cooling desk	None	None	None	*
Local airflow 1.6 m/s	-	None	None	*
Local airflow 2.2 m/s	-	-	None	None
Radiant cooling desk + local airflow 1.6 m/s	-	-	-	None
Radiant cooling desk + local airflow 2.2 m/s	-	-	-	-
32 °C				
No personal cooling	*	*	*	*
Radiant cooling desk	None	None	None	*
Local airflow 1.6 m/s	-	None	*	*
Local airflow 2.2 m/s	-	-	None	*
Radiant cooling desk + local airflow 1.6 m/s	-	-	-	None
Radiant cooling desk + local airflow 2.2 m/s	-	-	-	-

* *p* < 0.05, i.e., significant difference exists; None: No significant difference.

**Table 7:** Thermal comfort differences among personal cooling systems.

Conditions	Personal Cooling Strategies			
Radiant cooling desk	No	No	Yes	Yes
Local airflow 2 m/s	1.6	2.2	1.6	2.2
28 °C				
No personal cooling	None	None	None	None
Radiant cooling desk	None	*	None	*
Local airflow 1.6 m/s	-	None	None	None
Local airflow 2.2 m/s	-	-	*	None
Radiant cooling desk + local airflow 1.6 m/s	-	-	-	*
Radiant cooling desk + local airflow 2.2 m/s	-	-	-	-
30 °C				
No personal cooling	*	*	*	*
Radiant cooling desk	None	None	None	None
Local airflow 1.6 m/s	-	None	None	*
Local airflow 2.2 m/s	-	-	None	None
Radiant cooling desk + local airflow 1.6 m/s	-	-	-	*
Radiant cooling desk + local airflow 2.2 m/s	-	-	-	-
32 °C				
No personal cooling	*	*	*	*
Radiant cooling desk	*	*	None	*
Local airflow 1.6 m/s	-	None	*	*
Local airflow 2.2 m/s	-	-	*	*
Radiant cooling desk + local airflow 1.6 m/s	-	-	-	None
Radiant cooling desk + local airflow 2.2 m/s	-	-	-	-

* *p* < 0.05, i.e., significant difference exists; None: No significant difference.

**Table 8:** Air movement preference in the final votes.

Conditions	Average	Weaker Side	No Change	Stronger Side
28 °C				
No personal cooling	0.35	5%	55%	40%
Radiant cooling desk	0.3	5%	60%	35%
Local airflow 1.6 m/s	−0.5	55%	30%	15%
Local airflow 2.2 m/s	−0.95	75%	20%	5%
Radiant cooling desk + local airflow 1.6 m/s	−0.85	65%	35%	0
Radiant cooling desk + local airflow 2.2 m/s	−1.2	75%	25%	0
30 °C				
No personal cooling	1.35	0	10%	90%
Radiant cooling desk	0.6	0	50%	50%
Local airflow 1.6 m/s	−0.55	55%	35%	10%
Local airflow 2.2 m/s	−1.05	75%	20%	5%
Radiant cooling desk + local airflow 1.6 m/s	−0.55	55%	45%	0
Radiant cooling desk + local airflow 2.2 m/s	−1.05	80%	20%	0
32 °C				
No personal cooling	1.25	5%	5%	90%
Radiant cooling desk	1.1	0	20%	80%
Local airflow 1.6 m/s	0.2	20%	50%	30%
Local airflow 2.2 m/s	−0.2	45%	30%	25%
Radiant cooling desk + local airflow 1.6 m/s	−0.1	20%	70%	10%
Radiant cooling desk + local airflow 2.2 m/s	−0.65	50%	50%	0

**Table 9:** Results of discomfort symptoms obtained from the feedback.

Conditions	Fatigue ^1^	Dry ^2^	Humid ^2^	Stuffy Air	Dry Skin ^1^	Itchy Skin ^1^	Dry Lips ^1^	Dry Eyes ^1^	Thirsty ^1^	Dizzy ^1^
28 °C										
No personal cooling	15%	10%	15%	30%	10%	0	10%	10%	5%	0
Radiant cooling desk	5%	0	5%	30%	0	5%	0	0	0	0
Local airflow 1.6 m/s	15%	0	15%	15%	0	0	10%	15%	10%	0
Local airflow 2.2 m/s	10%	0	15%	15%	0	10%	0	15%	5%	10%
Radiant cooling desk + local airflow 1.6 m/s	5%	0	5%	15%	0	0	0	5%	0	5%
Radiant cooling desk + local airflow 2.2 m/s	0	0	5%	10%	0	5%	0	10%	0	10%
30 °C										
No personal cooling	0	5%	25%	40%	5%	5%	5%	5%	0	0
Radiant cooling desk	15%	5%	10%	30%	0	0	0	0	5%	0
Local airflow 1.6 m/s	5%	0	10%	25%	0	5%	5%	15%	0	5%
Local airflow 2.2 m/s	5%	0	5%	20%	0	0	0	10%	0	10%
Radiant cooling desk + local airflow 1.6 m/s	5%	0	0	20%	0	0	0	15%	0	15%
Radiant cooling desk + local airflow 2.2 m/s	5%	0	5%	20%	0	0	0	15%	0	15%
32 °C										
No personal cooling	10%	10%	40%	45%	10%	0	5%	0	0	0
Radiant cooling desk	5%	0	30%	50%	0	0	10%	0	5%	0
Local airflow 1.6 m/s	10%	10%	10%	35%	10%	0	5%	5%	0	5%
Local airflow 2.2 m/s	0	10%	10%	30%	10%	0	0	5%	0	5%
Radiant cooling desk + local airflow 1.6 m/s	0	0	5%	35%	0	0	5%	0	0	10%
Radiant cooling desk + local airflow 2.2 m/s	0	0	5%	25%	0	0	10%	0	0	20%

^1^ Not due to illness; ^2^ Indoor environment.

**Table 10:** Results of logistic regression under two modes.

Mode	Sample Size	Log Likelihood	Nagelkerke R^2^	Correct Percentage	Significant Variables	βn	Sig.	β0	Sig.
Free	60	23.512	0.740	95.0%	Effectiveness	5.533	0.000	−2.398	0.022
Charge	60	23.414	0.723	95.0%	Economy	5.460	0.000	−3.157	0.000

**Table 11:** Basic information of personal cooling systems in this study and previous ones.

Studies	Personal Cooling Systems	Temp. (°C)	Thermal Sensation	Cooling Energy (W)	*COP*	*CEP*_80%_^1^ (W/K)	*CEP*_90%_^2^ (W/K)
This study	Local air flow 1.6 m/s	28	−0.35	2	1	0.75 (−4 K)	0.75 (−4 K)
		30	−0.05	2	1		
	Local air flow 2.2 m/s	28	−0.55	3	1	0.92 (−6 K)	0.75 (−4 K)
		30	−0.15	3	1		
		32	0.7	3	1		
	Radiant cooling desk + local air flow 1.6 m/s	28	−0.6	74/2 *	8.5/1 *	3.86 (−6 K)	3.12 (−6 K)
		30	−0.1	90.2/2 *	8.5/1 *		
		32	0.3	140.5/2 *	8.5/1 *		
	Radiant cooling desk + local air flow 2.2 m/s	30	−0.4	100.4/3 *	8.5/1 *	3.44 (−6 K)	3.44 (−6 K)
		32	−0.05	136.9/3 *	8.5/1 *		
[23]	Radiant cooling desk	28	−0.1	89.9	8.5	3.64 (−6 K)	4.18 (−4 K)
		30	0.1	104.4	8.5		
		32	0.65	130.7	8.5		
[20]	Floor fans	30	0.45	8	1	2.25 (−4 K)	2.25 (−4 K)
		28	0.15	5	1		
[18]	Chairs with fans + USB fan	29	0.2	3.6/1.2 *	1/1 *	1.6 (−3 K)	1.6 (−3 K)
[37]	Four fans	28	0.31	41	1	20.5 (−2 K)	20.5 (−2 K)
[38]	Thermoelectric chair	29	0.5	45.5	1	15.17 (−3 K)	15.17 (−3 K)

* Corresponding to two types of personal cooling systems used at the same time, respectively; ^1^ The average *CEP* values of all conditions with thermal sensation within the range of −0.85 to +0.85, and the corresponding lowest *CP* value was in the brackets; ^2^ The average *CEP* values of all conditions with thermal sensation within the range of −0.5 to +0.5, and the corresponding lowest *CP* value was in the brackets.
